# IMPLEMENTATION OF DEMENTIA FRIENDS IN WASHINGTON STATE

**DOI:** 10.1093/geroni/igae098.3079

**Published:** 2024-12-31

**Authors:** Katie Zeitler

**Affiliations:** University of Washington, Seattle, Washington, United States

## Abstract

Dementia Friends (DF) is a global movement whose mission is to raise awareness about dementia and the challenges associated with its stigma. The purpose of this presentation is to trace the journey including the evidence supporting the implementation of DF in Washington State and describe next steps. The movement has 13.5 million participants worldwide and utilizes a train-the-trainer model to deliver information sessions to members of the community that discuss what dementia is, five key messages, and communication tips. From 2018-2019, DF was piloted in Washington with the support of the Dementia Action Collaborative. Data showed that Dementia Attitudes Scale scores improved after a DF session and that this improvement was maintained through the one-month follow up. The results from the pilot program led to the approval of funding from Washington state for the implementation of the program in 2022. The state lead for DF Washington, the UW Memory and Brain Wellness Center, implemented the program by partnering with Regional Lead Organizations to assist in deploying the train-the-trainer model to expand DF in their regions. Each Regional Lead Organization nominated a Regional Coordinator to oversee the DF program in their region and train new volunteer “DF Champions” to lead information sessions. Funding from the state and partnerships with Regional Lead Organizations have facilitated the expansion of DF program from 214 participants in 3 counties in 2019 to 2,170 participants in 24 counties in 2024. A key challenge for DF will be cultivating community partnerships in rural areas and underserved populations.

